# Evaluation of Cancer Stem Cell Markers CD133, CD44, CD24: Association with AKT Isoforms and Radiation Resistance in Colon Cancer Cells

**DOI:** 10.1371/journal.pone.0094621

**Published:** 2014-04-23

**Authors:** Sara Häggblad Sahlberg, Diana Spiegelberg, Bengt Glimelius, Bo Stenerlöw, Marika Nestor

**Affiliations:** 1 Section of Biomedical Radiation Sciences, Department of Radiology, Oncology and Radiation Science, Rudbeck Laboratory, Uppsala University, Uppsala, Sweden; 2 Section of Oncology, Department of Radiology, Oncology and Radiation Science, Uppsala University, Uppsala, Sweden; 3 Section of Otolaryngology and Head and Neck Surgery, Department of Surgical Sciences, Uppsala University, Uppsala, Sweden; 4 Science for Life Laboratory, Uppsala University, Uppsala, Sweden; Virginia Commonwealth University, United States of America

## Abstract

The cell surface proteins CD133, CD24 and CD44 are putative markers for cancer stem cell populations in colon cancer, associated with aggressive cancer types and poor prognosis. It is important to understand how these markers may predict treatment outcomes, determined by factors such as radioresistance. The scope of this study was to assess the connection between EGFR, CD133, CD24, and CD44 (including isoforms) expression levels and radiation sensitivity, and furthermore analyze the influence of AKT isoforms on the expression patterns of these markers, to better understand the underlying molecular mechanisms in the cell. Three colon cancer cell-lines were used, HT-29, DLD-1, and HCT116, together with DLD-1 isogenic *AKT* knock-out cell-lines. All three cell-lines (HT-29, HCT116 and DLD-1) expressed varying amounts of CD133, CD24 and CD44 and the top ten percent of CD133 and CD44 expressing cells (CD133^high^/CD44^high^) were more resistant to gamma radiation than the ten percent with lowest expression (CD133^low^/CD44^low^). The AKT expression was lower in the fraction of cells with low CD133/CD44. Depletion of AKT1 or AKT2 using knock out cells showed for the first time that CD133 expression was associated with AKT1 but not AKT2, whereas the CD44 expression was influenced by the presence of either AKT1 or AKT2. There were several genes in the cell adhesion pathway which had significantly higher expression in the *AKT*2 KO cell-line compared to the *AKT*1 KO cell-line; however important genes in the epithelial to mesenchymal transition pathway (*CDH1*, *VIM, TWIST1, SNAI1, SNAI2, ZEB1, ZEB2, FN1, FOXC2* and *CDH2)* did not differ. Our results demonstrate that CD133^high^/CD44^high^ expressing colon cancer cells are associated with AKT and increased radiation resistance, and that different AKT isoforms have varying effects on the expression of cancer stem cell markers, which is an important consideration when targeting AKT in a clinical setting.

## Introduction

Colorectal cancer is one of the most common diagnosed malignancies in the world. Several studies have identified subpopulations of colorectal cancer cells that are more resistant to cancer treatments such as chemotherapeutics and radiation [1], [2]. Successful treatment is dependent on the elimination of these highly resistant subpopulations, and not only the main tumor mass. These cells are often referred to as cancer stem cells or tumor-initiating cells, and several cell surface markers have been shown to be expressed in these cell populations [3]. CD133, CD44 and CD24 are three proposed stem cell markers in colorectal cancer, but discouragingly the distribution differs between patients and tumor cell lines [4]. It is therefore of great interest to understand their function and how the biomarkers interact with each other.

CD24 is a cell surface protein, which is anchored on the external side of the plasma membrane. It is thought to have an essential role in cell differentiation, and is also expressed in cells involved in the immune system, such as B-lymphocytes, where it positively regulates the proliferation of activated T cells. CD24 expression is also described in the central nervous system [5]. The distribution in colorectal cancer is under dispute, although previous studies have shown that between 50 and 68% of patients suffering from colorectal cancers expressed CD24 to a high extent [5], [6], and further that CD24 positive subpopulations from colon cancer cell-lines possess stem cell-like properties [7]. In contrast, tumor initiating cells from head-and-neck and breast cancer have been shown to be CD24 negative [8], [9].

CD133 (also called Prominin-1) is believed to be associated with tumorigenicity and progression of the disease. The up-regulation of CD133 in colorectal cancer correlates strongly with poor prognosis and synchronous liver metastasis [10], although the precise role and function of CD133 is unknown.

CD44 has a role in facilitation of cell to cell and cell-matrix interactions through its affinity for hyaluronic acid and is involved in cell-adhesion and the assembly of growth factors on the cell surface. CD44 is encoded by a single gene, including 20 exons. The standard form (referred to as CD44s) consists of exon 1–5 and 15–20. The variable exons are identified as v1–v10, respectively. The differential utilization of the 10 variant exons generates multiple CD44 variants (CD44v) with different combinations of variant exon products. Various isoforms of CD44 arise by insertion of one or more of the variant exons into the common backbone shared by all forms of CD44. The role of these variant isoforms is not fully understood, though some are believed to mediate a critical step in colon cancer metastasis [8], [11], [12]. CD44 can be co-immunoprecipitated with the family of ErbB receptor tyrosine kinases such as the epidermal growth factor receptor (EGFR) and it also interacts with HER2, HER3 and HER4 [8], [13]. EGFR is believed to play an important role in regulating and maintaining the cancer stem cells, mainly through downstream signaling via the Phospho-inositol 3 kinase (PI3K)/AKT pathway [14], [15].

AKT is a serine/threonine kinase with three different isoforms, AKT1, AKT2 and AKT3, expressed from three separate genes and activated by many stimuli, such as several growth factor receptors (for example EGFR), B and T cell receptors. It has a central role in many cellular functions responsible for proliferation, survival, growth, anti-apoptosis, glucose uptake, metabolism, angiogenesis and radioresistance [16]. AKT is also believed to be involved in the epithelial to mesenchymal transition (EMT) pathway which leads to increased motility, reduced intercellular adhesion, tumor progression and malignant transformation. The EMT pathway is therefore involved in cancer cell invasion and metastasis [17]. Inducers of EMT, such as receptor tyrosine kinase ligands or transforming growth factor beta (TGFβ), Wnt and Notch, triggers a cascade of cell-signaling which leads to the suppression of the cell adhesion protein E-cadherin. The process involves up-regulation of direct acting transcriptional repressors such as Snail, Slug, Forkhead box C2 and Zeb1, Zeb2 as well as Twist and E47 which indirectly repress E-cadherin. Other markers of EMT are N-cadherin, Vimentin and Fibronectin-1 which are expressed in mesenchymal cells [18]. EMT has also been shown to be involved in cancer stem-cells where colon cancer cells with a high expression of CD133/CD44 showed EMT after long-term culture [18], [19].

AKT has been proposed to be co-expressed with CD133, providing the CD133 expressing cell population with a higher resistance to chemotherapeutics, but the details about this interaction are not known [20], [21]. CD44 is believed to negatively correlate with AKT [22]. However, several studies have shown that AKT is instead phosphorylated when stimulating CD44 with ligand, causing a cell-survival effect [23]–[25], and it is probable that CD44 isoforms have a regulating role being able to both activate and suppress the activation of AKT. Radiation itself has also been shown to increase the expression of AKT, CD133, and reduce the expression of CD44 in colorectal cancer cells [26]. However, the importance of the different AKT isoforms on the CD133 or CD44 expression has not previously been studied.

We have recently shown that both AKT1 and AKT2 are important in the response to radiation [27]. Knocking-out either *AKT*1 or *AKT*2, or both simultaneously, increased the radiation sensitivity and the DNA double strand rejoining rate was impaired in the *AKT*1/2 KO cell-line. In the present study, we have investigated the differences in the expression patterns of CD133, CD24, CD44 and EGFR in three colon cancer cell-lines; HT-29, HCT116 and DLD-1. We have also analyzed the radiation sensitivity of the colon cancer cells sorted for CD133^high^/CD44^high^ and CD133^low^/CD44^low^ expression, and further investigated the influence of two AKT isoforms (AKT1, AKT2) on CD133 and CD44 expression, including CD44 splice variant isoforms. AKT3 is not expressed in the studied cell lines, and was therefore excluded. Furthermore, we validated the effect of AKT on gene expression in a large-scale transcriptomic analysis of multiple pathways.

## Materials and Methods

### Cell Culture

The colon cancer cell-lines HT-29 and HCT116 were acquired from The American Tissue Culture Collection (ATCC, Manassas, VA, USA), catalogue numbers ATCC CCl-221 and ATCC CCl-247, respectively. DLD-1 X-MAN isogenic cell-lines were obtained from Horizon Discovery Ltd with the different AKT isoforms genetically knocked-out, catalogue number HD-R00-001, HD-R00-002 and HD-R00-003. The cells were cultured in 75 cm^2^ culture flasks (Nunclon surface, Roskilde, Denmark) in McCoy’s 5A medium (Flow Irvine, UK) with 10% fetal bovine serum (Sigma Aldrich, St. Louis, USA), 2 mM L-glutamine, 100IU/ml penicillin and 10 µg/ml streptomycin (Biochrom Kg, Berlin, Germany). The cells were cultured in a humidified incubator with 5% CO_2_ at 37°C and trypsinized with trypsin-EDTA, 0.25% trypsin, 0.02% EDTA (Biochrom Kg, Berlin, Germany).

### Cell Sorting with Flow Cytometry

For flow cytometry analysis the cell-lines were harvested by using non-enzymatic cell dissociation solution (Sigma Aldrich, St. Louis, USA) or 0.25% trypsin, 0.02% EDTA, (Biochrom Kg, Berlin, Germany). After resuspension of the cells in cell-culture media, the cells were counted and washed in PBS with 0.5% BSA and collected by centrifugation. The cells were incubated for 10 to 30 minutes with the labelled antibodies, see Table 1. Following the antibody labeling the cells were washed in PBS with 0.5% BSA before flow cytometric analysis was carried out on a SORP BD LSRII (Becton Dickinson Biosciences, San Jose, USA). Dying and dead cells were stained with propidium iodide and excluded from analysis. Duplicates and dead cells were also excluded by gating with FSC and SSC. For cell-sorting for clonogenic assay the FACSVantage SE DiVa or FACSAriaIII Cell sorter (Becton Dickinson Biosciences, San Jose, USA) were used.

**Table 1:** Antibodies used for flow cytometry experiments.

Marker	Antibody
**CD133**	PE conjugated Anti-human CD133 (eBioscience, San Diego, USA)
	PE or APC conjugated anti-human CD133/1 (Miltenyi Biotech, Germany)
	PE conjugated anti-human CD133/2 (AC133) (Miltenyi Biotech, Germany)
**CD44**	APC conjugated anti-human CD44 (eBioscience, San Diego, USA)
	APC conjugated anti-human CD44 (Miltenyi Biotech, Germany)
**CD24**	FITC conjugated anti-human CD24 (eBioscience, San Diego, USA)
	FITC conjugated anti-human CD24 (Miltenyi biotech, Germany)
**CD44v3**	PE conjugated anti CD44v3 (R&D systems, Minneapolis, USA)
**CD44v4**	FITC conjugated mouse anti-human CD44v4 (AbD serotec, Oxford, UK).
**C44v4/5**	PE conjugated anti CD44v4/5 (R&D systems, Minneapolis, USA)
**CD44v6**	FITC conjugated mouse anti-human CD44v6 (AbD serotec, Oxford, UK).
**CD44v7**	FITC conjugated mouse anti-human CD44v7 (AbD serotec, Oxford, UK).
**CD44v7/8**	FITC conjugated mouse anti-human CD44v7/8 (AbD serotec, Oxford, UK).
**EGFR**	FITC or APC conjugated EGFR (AbCam, Cambridge, UK)
**Fluorophore**	**Isogenic control antibody**
**PE**	Mouse IgG2B (R&D systems, Minneapolis, USA or Miltenyi biotech, Germany)
	Mouse IgG1 (eBioscience, San Diego, USA)
**FITC**	Mouse IgG1 (AbD serotec, Oxford, UK or Miltenyi biotech, Germany)
	Mouse IgG2B (AbD serotec, Oxford, UK)
**APC**	Rat IgG2b (eBioscience, San Diego, USA)
	Mouse IgG2B (Miltenyi biotech, Germany)

### Cell-cycle Analysis

Cells were fixated with 70% ethanol, 30% PBS and kept in −20°C for at least 24 hours. Cells were centrifuged for 10 minutes, 2000G in 4°C and washed twice with PBS before incubation with 5 µg propidium iodine/0.1% NP-40 (Sigma Aldrich, St. Louis, USA) in PBS together with 5 µg RNase (Sigma Aldrich, St. Louis, USA) for 30 minutes at room temperature. Analysis was made with flow cytometry (BD LSRII Biosciences).

### Clonogenic Assay

The association between the expression of CD133 and CD44 and radiation sensitivity was evaluated by sorting and collecting CD133^high^/CD44^high^ and CD133^low^/CD44^low^ expressing cells. The cell number after sorting was determined with a cell counter (TC20 Automated cell counter, Biorad Life science, Hercules, CA, USA)), and a certain amount of cells was pre-plated in 25 cm^2^ tissue culture flasks. The following day the cells were exposed to externally applied radiation using a ^137^Cs source (Best Theratronics Gammacell 40 Exactor, Springfield, USA). After an incubation time period of 8–14 days the cells were washed in PBS and fixed with 99.5% ethanol for 5–10 min. The colonies were stained with Mayer’s Haematoxylin (Histolab Products AB, Västra Frölunda, Sweden) for 20–30 min and thereafter rinsed in water. Colonies with more than 50 cells per colony were counted and the plating efficiency (PE = number of colonies in untreated cells/number of cells seeded) and the survival fraction (SF = number of colonies in treated cells/number of cells seeded × PE) were calculated and plotted.

### Statistical Analyses

Flow cytometry analyses were evaluated using BD FACSDiva software 7.0 (BD Biosciences). The clonogenic assay data was processed with Microsoft office Excel 2007 (Microsoft, Redmond) and graphs were plotted and analyzed with one-way ANOVA in GraphPad Prism 5 (GraphPad Software, San Diego, USA). A significance level of 95% was used. This analysis evaluated whether the survival fractions of the radiated CD133 and CD44 sorted cells were significantly different from each other for each radiation dose.

### Western Blot

Cells were cultivated in 3 cm Petri dishes for at least three doubling times prior lysation or three separate cell-cultures of DLD-1 were sorted by flow cytometry (see above) and the volume of lysis buffer was adjusted to the number of cells collected in each vial. Lysates were prepared post-treatment by washing the cells with ice-cold PBS followed by addition of 10 000 000 cells/ml lysis buffer containing 1% Tween-20, 20 mM Tris (pH 8.0), 137 mMNaCl, 10% glycerol, 2 mM EDTA, 1 mM activated sodium orthovanadate (Sigma Aldrich, St. Louis, USA) and protease inhibitor cocktail (P8340, Sigma Aldrich, St. Louis, USA) and incubation on ice for 30 min. Lysates were centrifuged for 10 min in 4°C. The supernatant was transferred to new tubes and the pellet discarded. The protein concentration of the lysate was determined by BCA protein assay (Pierce). Equal amounts of protein were loaded on a Tris-Acetate 3–8% SDS PAGE gel (Life Technologies, Carlsbad, CA) and afterwards transferred to a nitrocellulose membrane (Millipore) by wet blotting. The nitrocellulose membrane was blocked for 1 h in 5% BSA, PBS and then incubated with the primary antibody overnight at 4°C. Antibody specific for CD133/1 (W6B3C1) was from Miltenyi biotech (Heidelberg, Germany) and CD44 (103014) from Biolegend (San Diego, CA, USA). Antibodies against FoxO3a (2497), phospho-FoxO (2599) and GSK-3Beta (9315) and phospho-GSK3B (9323) were all from Cell Signaling Technology (Beverly, MS, USA). AKT1 (sc55523 and AKT2 (sc5270) were from Santa Cruz Biotechnology (Santa Cruz, CA, USA). Antibody against β-actin (A5441) was from Sigma-Aldrich (St. Louis, USA). After washing in PBS with 1% Tween-20, the membrane was incubated with horseradish peroxidase-labeled secondary antibody (626520 and 656120) (Invitrogen, Camarillo, CA, USA) or (405405) (Biolegend, San Diego, CA, USA) for 1 h at room temperature. Immuno-reactive bands were visualized in a CCD camera (SuperCCD HR, Fujifilm, Japan) after treatment with Immobilon electro-chemiluminescent solution (Millipore, Billerica, MA, USA) for 5 min.

### Microarray Expression Analysis

Two separate passages of DLD-1 parental, *AKT*1 KO, *AKT*2 KO and *AKT*1/2 KO cells were cultured to 70% confluence and RNA was extracted (RNeasy mini-prep, Qiagen, Valencia, CA, USA) RNA concentration was measured with ND-1000 spectrophotometer (NanoDrop Technologies, Wilmington, DE) and RNA quality was evaluated using the Agilent 2100 Bioanalyzer system (Agilent Technologies Inc, Palo Alto, CA). 250 nanograms of total RNA from each sample were used to generate amplified and biotinylated sense-strand cDNA from the entire expressed genome according to the GeneChip WT PLUS Reagent Kit User Manual (P/N 703174 Rev 1 Affymetrix Inc., Santa Clara, CA). GeneChip HTA Arrays (GeneChip Human Transcriptome Array 2.0) were hybridized for 16 hours in a 45°C incubator, rotated at 60 rpm. According to the GeneChip Expression Wash, Stain and Scan Manual (PN 702731 Rev 3, Affymetrix Inc., Santa Clara, CA) the arrays were then washed and stained using the Fluidics Station 450 and finally scanned using the GeneChip Scanner 3000 7G.

### Microarray Data Analysis

The raw data was normalized in the free software Expression Console provided by Affymetrix (http://www.affymetrix.com) using the robust multi-array average (RMA) method first suggested by Li and Wong in 2001 [28], [29]. Subsequent analysis of the gene expression data was carried out in the freely available statistical computing language R (http://www.r-project.org) using packages available from the Bioconductor project (www.bioconductor.org). In order to search for the differentially expressed genes between parental and the *AKT* KO groups an empirical Bayes moderated t-test was then applied, using the ‘limma’ package [30]. To address the problem with multiple testing, the p-values were adjusted using the method of Benjamini and Hochberg [31]. The normalized data was further evaluated using DAVID Bioinformatic resources 6.7 to together with Kyoto encyclopedia of genes and genomes (KEGG) pathway database (http://www.genome.jp/kegg/pathway.html) to functionally classify and cluster the genes related to epithelial to mesenchymal transition pathways [32], [33].

### Confirmation of *AKT*1 and *AKT*2 Knock-out with PCR

Total RNA was isolated from DLD-1 parental, *AKT*1 KO, *AKT*2 KO and *AKT*1/2 KO cells with RNeasy mini kit (Qiagen, Valencia, CA, USA). cDNA was synthesized from 0.1 µg total RNA using RevertAid H Minus First Strand cDNA Synthesis Kit with random hexamer primers (Thermo Scientific, Waltham, MA, USA) and PCR was performed with Taq DNA Polymarese (Thermo Scientific, Waltham, MA, USA) with primers against AKT1 (fwd: AGGCTCCCCTCAACAACTTC, rev: CTCCTCCTCCTCCTGCTTCT) or AKT2 (fwd: GGTGCCTCCTGCATGTCC, rev: CCTCTCGGTCTTCATCAGC).

### Transfection with siRNA against AKT1 in DLD-1 Parental Cells

The cells were transfected with siAKT1 silencer (ambion by Life Technologies, Carlsbad, CA) with Lipofectamine 2000 (Life Technologies, Carlsbad, CA) (sense: 5′ GCGUGACCAUGAACGAUUtt and antisense: 5′AACUCGUUCAUGGUCACGCGG). The transfected cells were incubated in 37°C in a CO_2_ incubator for 48 hours before analyzing the CD133, CD44 and CD24 expression on flow cytometry, see above.

### Re-activation of AKT1 or AKT2 in DLD-1 *AKT*1/2 KO Cells

DLD-1 *AKT*1/2 KO cells cultured to a confluence of 30–50% in antibiotic-free McCoys cell media (Sigma Aldrich, St. Louis, USA) for 24 hours before transfection. The cells were transfected with pcDNA3 myr HA AKT1 and pcDNA3 myr HA AKT2 plasmids kindly provided by William Sellers (Dana Farber Cancer Institute, Boston, MA, USA) through Addgene (Cambridge, MA). Lipofectamine 2000 and OptiMEM were from Life technologies (Carlsbad, CA). The transfected cells were incubated in 37°C in a CO_2_ incubator for 72 hours before further analyzing the CD133, CD44 and CD24 expression by flow cytometry, see above.

## Results

### CD133, CD44, CD24 and EGFR Expression in Colon Cancer Cell-lines

**Figure 1:**
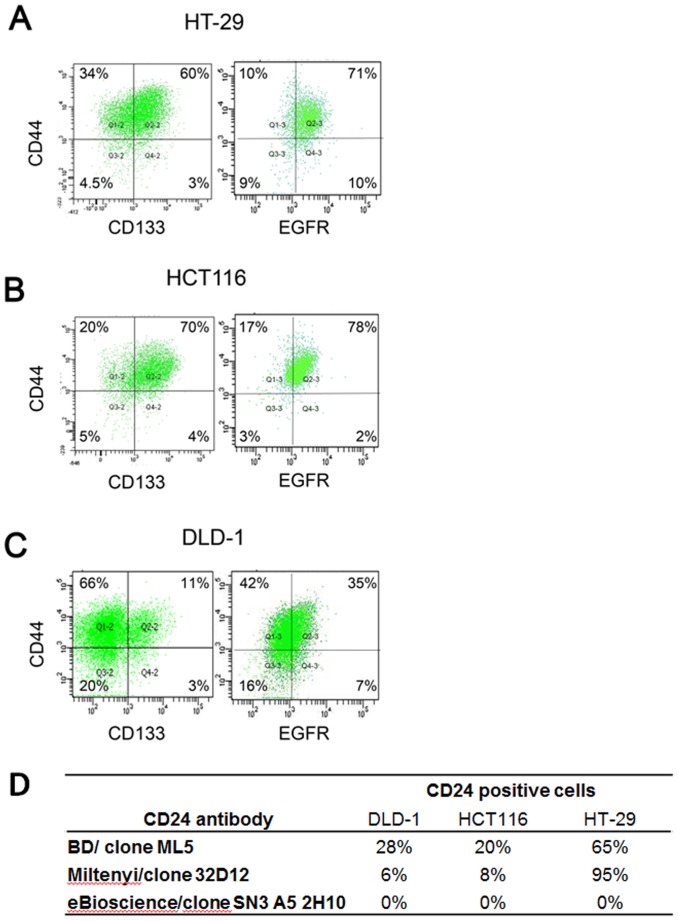
Expression of CD133, CD44, CD24 and EGFR in three colon cancer cell-lines. A) HT-29, HCT116 and DLD-1. The expression patterns in the dotplots are from one representative flow cytometer experiment. The grid demonstrates the margin between high and low expression of the protein defined by isotype controls. B) The expression of CD24 positive cells in flow cytometry depend on the anti-CD24 antibody. The table shows the percent of CD24 positive cells using three different CD24 antibodies.

The CD133, CD44, CD24 and EGFR expression in three colon cancer cell-lines was analyzed with flow cytometry, see Figure 1A. There was a difference in the expression of CD133, CD44, CD24 and EGFR between the cell-lines. CD44 was displayed as one population stretching from low to high expression in all three cell-lines. The detection of CD24 expression was dependent on the anti-CD24 antibody. The highest expression of CD24 was seen in the HT-29 cells with 95% positive cells using the CD24 antibody from MACS), see figure 1B. CD133 was expressed in the majority of the HCT116 and HT-29 cells, whereas only 14% of the DLD-1 cells were positive for CD133. All three cell-lines expressed EGFR. Around 80% of HT-29 and HCT116 where positive for EGFR whereas 40% were positive in DLD-1.

### Radiosensitivity of CD133^high^/CD44^high^ and CD133^low^/CD44^low^ Expressing Cells

**Figure 2:**
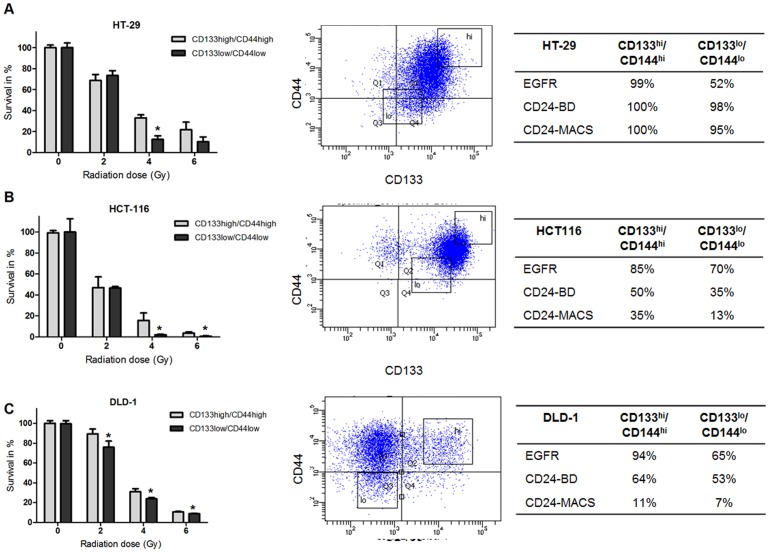
Radiation sensitivity of CD133/CD44 sorted cells. Clonogenic assay of A) HT-29, B) HCT116 and C) DLD-1 cells. The top and bottom 10 percent of CD133 and CD44 expressing cells were sorted by flow cytometry (CD133^high^/CD44^high^ or CD133^low^/CD44^low^) and the radiation sensitivity was analyzed using clonogenic assays. The controls of both fractions were normalized and set to 100% survival. The error bars represent the standard error of the mean from at least two separate experiments with triplicate samples. A higher resistance to radiation in the CD133^high^/CD44^high^ population compared to the CD133^low^/CD44^low^ population was observed for all cell-lines. These differences were statistically significant at all radiation doses (2, 4 and 6 Gy) for DLD-1 cells (Figure 2C), at 4 and 6 Gy for HCT116 cells, and at 4 Gy for HT-29 cells (Figure 2A and B) with a P-value of <0.05 (one-way ANOVA). The percentage of cells positive for EGFR, CD24 with BD bioscience antibody or CD24 with Miltenyi biotech/MACS were analyzed with flow cytometry.

The association between the expression of CD133, CD44 and radiation sensitivity was evaluated by sorting and collecting CD133^high^/CD44^high^ and CD133^low^/CD44^low^ expressing cells, followed by exposure to externally applied gamma radiation, and further analyzed with clonogenic assays, see Figure 2. A higher resistance to radiation in the CD133^high^/CD44^high^ population compared to the CD133^low^/CD44^low^ population was observed for all cell-lines. These differences were statistically significant at all radiation doses (2, 4 and 6 Gy) for DLD-1 cells, see Figure 2C, at 4 and 6 Gy for HCT116 cells, and at 4 Gy for HT-29 cells, see Figure 2A and B, with a P-value of <0.05 (one-way ANOVA). There was no or a small difference in the expression of CD24 in the CD133^high^/CD44^high^ and CD133^low^/CD44^low^ population, except in HCT116 where there were less positive CD24 cells in the CD133^low^/CD44^low^ fraction. The numbers of EGFR positive cells in the sorted fractions were almost the double in the CD133^high^/CD44^high^ compared to the CD133^low^/CD44^low^ fraction in HT-29 and DLD-1 cells whereas in HCT116 there was only a small difference in the EGFR expression between the fractions. The radiation sensitivity for unsorted cells is shown in Figure S1.

### Expression of AKT in CD133^positive^/CD44^positive^ and CD133^negative^/CDD44^negative^ Population in DLD-1

**Figure 3:**
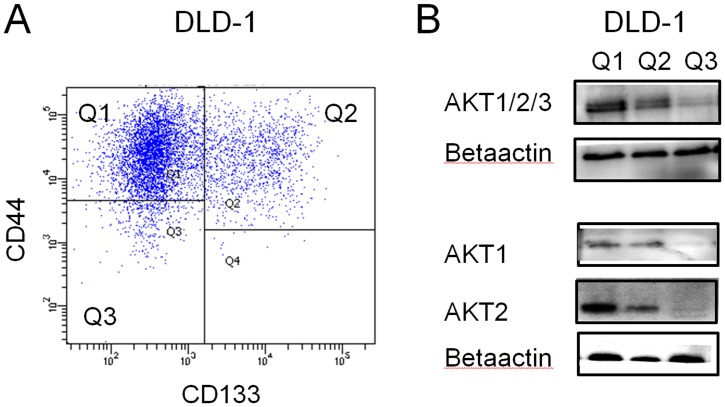
AKT expression in CD133/CD44 sorted cells. A) DLD-1 cells were sorted by flow cytometry and different populations with CD44^positive^/CD133^negative^ (Q1), CD44^positive^/CD133^positive^ (Q2), CD44^negative^CD133^negative^ (Q3), were collected. B) The sorted cells were further analyzed with western blot for total AKT, AKT1 or AKT2 and betaactin expression.

The different populations of CD133^positive^/CD44^positive^, CD133^negative^/CD44^positive^ and CD133^negative^/CD44^negative^ in DLD-1 cells were sorted, collected and further analyzed for expression of AKT with western blot, see Figure 3A and B. The expression of total AKT was lower in the CD133^negative^/CD44^negative^ population compared to the population positive for CD44 and/or CD133. Similar pattern was seen for AKT1 and AKT2, see Figure 3B.

### Influence of AKT Isoforms on the Expression of CD24, CD133 and CD44

**Figure 4:**
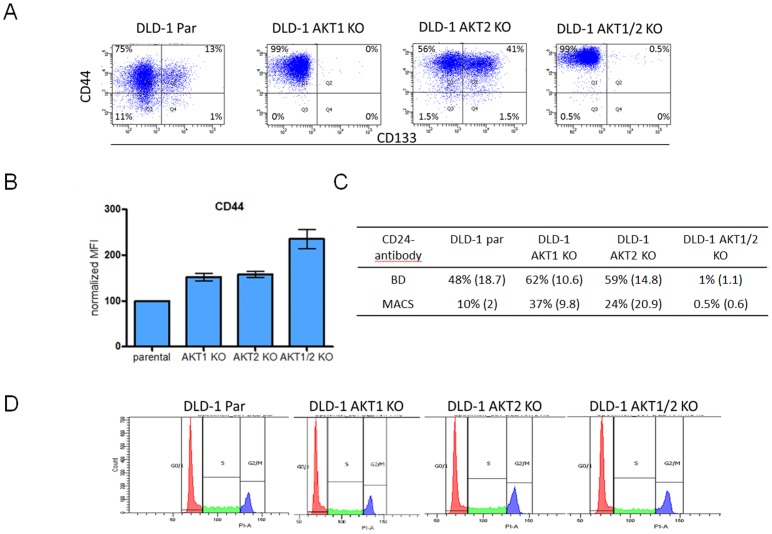
Flow cytometry analysis of the expression of CD133, CD24 and CD44 in the colon cancer cell-line DLD-1 with its isogenic knock-out cell-lines of *AKT*1, *AKT*2 and *AKT*1/2. A) In the parental cells, approximately 10% of the cells were CD133 positive cells. However, in the *AKT*1 and *AKT*1/2 knock-outs, the CD133 positive cells were reduced to 0.3 and 0.1% respectively. This was not seen in the *AKT*2 knock-out cell-line, where 33% of the cells were positive for CD133. B) The mean fluorescent intensity of CD44 normalized to the DLD-1 parental cell-line increased to 150% in *AKT*1 KO, 160% in *AKT*2 KO and 300% in *AKT*1/2 KO cell-line. The error bars represent the standard deviation (SD) from at least two experiments. C) The percent of CD24 positive cells analyzed with two different CD24 antibodies from BD Biosciences and Miltenyi/MACS in flow cytometry. The standard deviations are from repeated experiments. D) Cell-cycle distribution in DLD-1 parental, *AKT*1 KO, *AKT*2 KO and *AKT*1/2 KO cells.

Since the AKT expression was different in the sorted CD133^positive^/CD44^positive^and CD133^negative^/CD44^negative^ populations, the influence of AKT isoforms was evaluated using the colon cancer cell-line DLD-1 and the *AKT*1, *AKT*2 and *AKT*1/2 isogenic knock-out cell-lines, see Figure 3AB, and confirmation of knock-outs in Figure S2. The mean fluorescent intensity (MFI) of CD44 expression was increased from 100% in parental to 150% in *AKT*1 KO and *AKT*2 KO and 250% in *AKT*1/2 KO cell-line, see Figure 4A and B. Furthermore, the CD133 expression was reduced when AKT1 was knocked-out as seen in the *AKT*1 KO as well as in the *AKT*1/2 KO cell-line. However, single knock-out of *AKT*2 as seen in the *AKT*2 KO cell-line did not reduce the level of CD133, see Figure 4A. The CD24 expression was completely abolished in the *AKT*1/2 KO but increased in the single *AKT*1 or *AKT*2 KO cell-lines see Figure 4C. The influence of AKT isoforms on the expression of CD24, CD133 and CD24 were further verified with siRNA against AKT1 and by reintroduction of AKT1 or AKT2 in the DLD-1 *AKT*1/2 KO cell-line, see figures S3 and S4. Additionally, we confirmed that there was no difference in the cell-cycle distribution between the cell-lines, see Figure 4D.

### Influence of AKT Isoforms on the Expression of CD44 Variant Isoforms

The majority (98–100%), of DLD-1, HCT116 and HT-29 cells were positive for CD44, detected with an antibody which detects all variants of CD44. The expression pattern of CD44 variant isoforms v3, v4/5, v6, v7 and v7/8 were further investigated in the DLD-1 parental and *AKT*1/2 KO cell-lines see Table 2. Only small amounts (∼1–6%) of cells were positive for the CD44 variant isoforms (expression in single, live cells), and with no significant changes in expression levels between AKT proficient and deficient DLD-1 cell-lines. In the case of CD44v7, which had a higher detectable level, the expression was slightly reduced in the *AKT*1/2 KO cells compared to parental.

**Table 2:** The expression of the CD44 variant isoforms v3, v4, v4/5, v6, v7 and v7/8 in DLD-1 parental and *AKT*1/2 KO presented as the mean fraction (min-max value) of live cells from at least two flow cytometry experiments.

CD44 variant isoform	DLD-1*	DLD-1 *AKT* 1/2 KO*
**v3**	1.8 (0.6–3.5)	1.8 (0.6–2.8)
**v4/5**	0.2 (0.1–0.2)	0.8 (0.4–1.5)
**v6**	0.5 (0.4–0.7)	0.6 (0.4–0.9)
**v7**	6.0 (5.8–6.2)	4.0 (3.8–4.1)
**v7/8**	0.5 (0.1–0.5)	0.4 (0.3–0.4)

The expression of CD44 standard variant was around 100%.

^*^Mean fraction (min-max).

### Biochemical Analyses of DLD-1 *AKT* Knock Out Cell-lines

**Figure 5:**
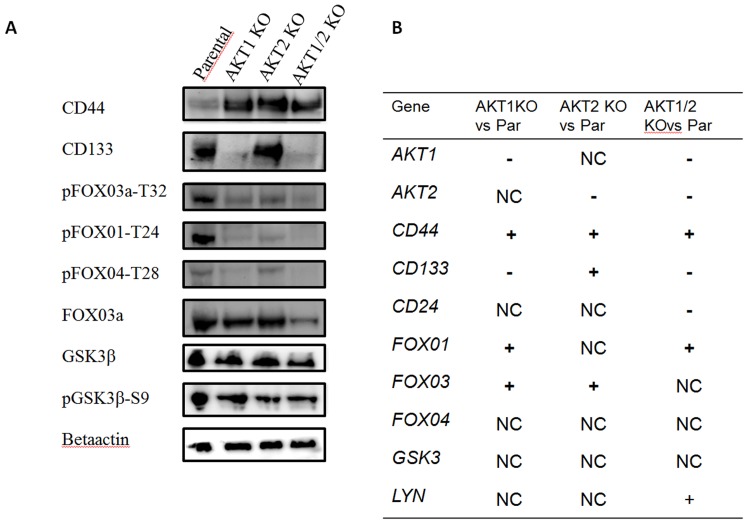
Protein and gene expression in DLD-1 parental, *AKT*1 KO, *AKT*2 KO, and *AKT*1/2 KO cells. A) Protein expression of CD44, CD133, phospho-FOXO, total FOX03a, phospho-GSK3β and total GSK3β from western blot analysis. B) Gene expression, up-regulation (+), down-regulation (−) or not changed (NC), of CD44, CD24, CD133, FOX01, FOX03, FOX04, GSK3β and LYN in DLD-1 *AKT*1 KO, *AKT*2 KO or *AKT*1/2 KO cells in comparison with DLD-1 parental cells.

Western blot analysis further evaluated the protein expression of CD133 and CD44 as well as Fox0 and GSK3β and their influence by AKT isoforms. The expression of CD44 was up-regulated in the *AKT*1 KO, *AKT*2 KO and *AKT*1/2 KO cell-lines compared to parental and the CD133 expression was reduced in *AKT*1 KO and *AKT*1/2 KO cell-lines, but not in the *AKT*2 KO cell-line as seen in the flow cytometry analysis. The *AKT*1, A*KT*2 and *AKT*1/2 KO cell-lines had a reduced expression of phosphorylated Fox01 and Fox03a as well as a reduced expression in total Fox03a in *AKT*1/2 KO cell-line. However, there were no differences in the expression of phosphorylated (S9) or total GSK3β between the *AKT* KO cell-lines, see Figure 5A.

### Differences in Gene Expression in the DLD-1 AKT Isoforms Knock-out Cell-lines

Gene expression analysis was performed to further investigate the differences between the isogenic *AKT* isoform knock-out cell-lines see Figure 5B. Genes were considered significantly up- or down- regulated with ratios ≥1.5 fold and with p<0.05. The knock-out of the *AKT*1 and *AKT*2 isoforms was confirmed in the DLD-1 cell-lines and the gene expression of CD44 and CD133 confirmed results from the flow cytometry and western blot analysis. CD44 was up-regulated in the *AKT*1, *AKT*2 and *AKT*1/2 KO cell-lines compared to parental cell-line and CD133 was up-regulated in *AKT*2 KO but down-regulated in *AKT*1 and *AKT*1/2 KO cell-lines. The CD24 expression was lower in the *AKT*1/2 KO cell-line compared to parental however; there was no difference in the *AKT*1 or *AKT*2 KO cell-lines. FOXO1 was up-regulated in *AKT*1 KO and *AKT*1/2 KO cell-line, while FOXO3 was only up-regulated in single *AKT*1 KO and *AKT*2 KO cell-lines. There was no difference in FOXO4 expression between the cell-lines. LYN was up-regulated only in the *AKT*1/2 KO however, there was no difference in the expression of GSK3β between the cell-lines, see Figure 5B.

### Evaluation of Differences in Epithelial to Mesenchymal Transition, Notch, Wnt and Cell Adhesion Pathways between *AKT*2 KO and *AKT*1 KO Cell-lines

One marker for epithelial to mesenchymal transition (EMT) is the reduction of cell adhesion. There were several genes in the cell adhesion pathway (*CLDN1, ITGB8, NEO1* and *PVRL3*) which had significantly higher expression in the *AKT*2 KO cell-line compared to the *AKT*1 KO cell-line. Furthermore, induction of EMT involves Notch and Wnt and tyrosine kinase receptors. Genes in the Notch pathway *NFKBIE* and *HES1* had a higher expression in the *AKT*2 KO cell line and two genes in the Wnt-pathway, *PRKCA* and *WNT5A* were differently expressed. *PRKCA* was up-regulated and *WNT5A* was down-regulated in the *AKT*2 KO cell-line. Important genes in the epithelial to mesenchymal transition (EMT) pathway are *CDH1* (encoding E-cadherin), *VIM, TWIST1, SNAI1, SNAI2, ZEB1, ZEB2, FN1, FOXC2* and *CDH2* (encoding N-cadherin) [19]. However, none of these genes had any significant differences (≥1.5 fold and with p<0.05) in the expression between the *AKT*2 and *AKT*1 KO cell-lines, see Table 3.

**Table 3:** The log fold change of genes involved in important pathways in cancer in DLD-1 *AKT*2 KO versus *AKT*1 KO.

Gene Symbol	Gene Description	Pathway	log fold change	p-value
PRKCA	protein kinase C, alpha	WNT	0.56	2.05E-04
WNT5A	wingless-type MMTV integration site family 5A	WNT	−1.34	1.96E-10
HES1	hairy and enhancer of split 1	Notch	0.77	1.34E-05
JAG1	jagged 1	Notch/EMT	0.74	2.11E-07
ALCAM	activated leukocyte cell adhesion molecule	CAMs	0.56	1.01E-04
CLDN1	claudin 1	CAMs	1.22	1.49E-08
ITGB8	integrin, beta 8	CAMs	1.25	6.96E-07
NEO1	neogenin 1	CAMs	1.61	7.34E-10
PVRL3	poliovirus receptor-related 3	CAMs	2.32	4.70E-11

Genes related to the WNT, Notch and cell adhesion molecules (CAMs) pathways were differently expressed.

## Discussion

Identification of combinations of biomarkers could be important for improved cancer detection, individualized cancer therapy, and better tumor control. This was recently pinpointed in a study using material from colorectal cancer patients, which showed that a combined expression of high CD133/CD44 was useful to identify putative colorectal cancer stem cells and tumors with a poor prognosis [34]. In addition, CD133/CD44 highly expressing populations of cancer cells have been shown to be invasive *in vitro* and are responsible for metastases *in vivo* in mice [19], [35]. Clinical studies have also shown the importance of CD133 or CD44v6 and response to radiochemotherapy [36]. The detection of these surface markers are highly dependent on the binding epitopes of the antibodies and the method used, i.e. flow cytometry, western blot, immunohistochemistry or qPCR, which have resulted in ambiguous results [37]. We have therefore confirmed our results with at least two types of antibodies against the different cancer stem cell markers. In this study we first demonstrate, in accordance with previous studies [38], [39], that the three colon cancer cell-lines were CD44 positive (∼90%) with a broad intensity spectrum in the same cell line, from low to high CD44 expressing cells. The CD133 expression was varying in the three cell-lines from 14% in DLD-1 to 74% and 63% in HCT116 and HT-29 respectively. The analysis of the CD24 expression was dependent on the anti-CD24 antibody. The antibody from ebioscience (clone SN3 A5-2H10) did not show any expression, whereas the one from MACS/Miltenyi (clone 32D12) displayed a CD24 expression in 28%, 20% and 65% of the DLD-1, HCT116 and HT-29 cells respectively. The antibody from BD (clone ML5) instead presented a CD24 expression in 6%, 8% and 95% of the DLD-1, HCT116 and HT-29 cells respectively. This demonstrates the difficulties in comparing results from different clones and epitopes, and emphasizes the importance of verifying results with an additional antibody. We have also shown that EGFR was highly expressed in HT-29 and HCT116, and moderately in DLD-1 cells and that the EGFR expression was higher in the CD44 or CD133 positive fraction. The expression of these surface markers was also confirmed to be independent of cell-cycle phase.

The radioresistance of cancer stems cells has been supported by several research groups in glioma [40]–[43], head and neck [44], breast [45], [46], pancreatic [47], and colorectal cancer [48]. A recent meta-analysis study has shown that CD133 expression is a good prognostic marker in colorectal cancer, where a high expression of CD133 correlates with a worse prognosis [49]. On the other hand, a contradictive study by Dittfeld *et al.* [50] proposed that CD133 expression in the colon cancer HCT116 cell line was not selective for radioresistance. However, it should be noted that Dittfeld *et al.* used a different CD133 antibody clone (CD133/1, Miltenyi) compared to our study which has used CD133/2 (Miltenyi) or CD133/TMP4 (eBioscience, San Diego, CA, USA) for sorting and the CD133/1 antibody (Miltenyi) for western blot. Previous studies which confirm the CD133 marker as a cancer stem cell marker have used a dual sorting method using both the CD133/1 and CD133/2 clone [51], [52].

In our study, we show that the colon cancer cell-lines sorted for CD44^high^/CD133^high^ and CD44^low^/CD133^low^ populations had a significant difference in survival after exposure to radiation. Cells with a CD44^high^/CD133^high^ expression demonstrated a higher radioresistance compared to CD44^low^/CD133^low^ cells. The resistance to radiation in CD44^high^/CD133^high^ expressing cells indicates that these cells are able to avoid cell-death and continue to grow and proliferate despite the exposure to radiation. We also show that the cell population positive for CD44 and/or CD133 had a higher expression of total AKT compared to cells with a low expression of both CD44 and CD133. This is in line with a recent study, in which we showed that AKT (both AKT1 and AKT2) interacts with DNA repair protein DNA-PKcs and that the knock-out of either *AKT*1, *AKT*2 or dual knock-out of both isoforms, increased the sensitivity to radiation and impaired the DNA double strand break rejoining rate [27].

The AKT-pathway is involved in anti-apoptosis, cell proliferation and resistance to radiation [53]. There are several proteins affected downstream of AKT which mediates these functions such as Forkhead family of transcription factors (FoxO) and glycogen synthase kinase (GSK3β) [54]. Phosphorylation of FoxO (Thr24- FoxO1, Thr32-FoxO3, and Thr28- FoxO4) leads to degradation of FoXO through ubiquitination which will lead to progression through cell cycle and proliferation [55]. In the DLD-1 *AKT* knock-out cell-lines the phosphorylation of FoxO1 and FoxO3 was reduced independent of AKT isoform, whereas the gene expression of *FOXO1* was significantly up-regulated in the *AKT*1 and *AKT*1/2 KO cells while *FOXO3* was up-regulated in both *AKT1* KO and *AKT*2 KO but not in the *AKT*1/2 KO cell-line. This suggests that the different AKT isoforms regulate the expression of FoxO differently whereas the expression or phosphorylation of FoxO4 was not dependent on AKT. Recent studies have shown that the effect of AKT signaling on cancer stem cells is mediated by β-catenin. When β-catenin is bound in a complex with APC, Axin and GSK3β it will be degraded. When Wnt activates the Frizzled receptor the complex is disrupted and β-catenin is able to translocate to the nucleus and act as a transcription factor promoting the expression of several genes associated with progression and invasion of the disease such as EMT. *CD44* is one of the genes activated by β-catenin [56], and CD133 positive colon cancer cells have a higher β-catenin expression level [57]. AKT induces the nuclear translocation β-catenin through the phosphorylation of GSK3β [14], [58]. In our study, the total expression or phosphorylation of GSK3β was however not affected by the knock-out of *AKT* in DLD-1 cells, suggesting that AKT mediates through other pathways. Mutations in *APC* stabilize β-catenin and cause a constitutive activation of Wnt signaling. HT-29 and DLD-1 have mutations in the tumor suppressor *APC,* and HCT116 has an activating mutation in one of the β-catenin alleles [59], [60].

There is a discrepancy among published findings regarding the role of AKT in promoting cancer stem-cells. This is probably due to the different experimental models and factors associated with mouse models vs. clinical studies, knockdown vs. over-expression, and *in vitro* vs. *in vivo* studies. However, supporting studies have shown that AKT inhibition by the PI3K inhibitor LY-294002 or the PI3K/mTor inhibitor NVP-BEZ can down-regulate the CD133 expression in colorectal cancer cell-lines [61] and prostate cancer [62]. Also, a study by Ma *et al*. indicated that hepatocellular carcinoma cells sorted for high and low CD133 expression had a higher activity of AKT in the CD133^high^ cells [20]. However, the influence of AKT isoforms on the expression of CD133 has not previously been addressed. In the present study, we show for the first time that the CD133 expression was AKT isotype dependent, since the knock-out of *AKT*1, but not *AKT*2 knock-out, reduced the expression of CD133. This expression pattern of CD133 was shown with flow cytometry, and further confirmed with western blot and mRNA expression analysis of *AKT* knock-out DLD-1 cell-lines.

Both AKT and CD44 are suggested to a have a dual role of both activating and inhibiting oncogenic signaling. AKT regulates, and is itself regulated, through several complex signaling pathways and is also dependent of factors such as cell-type and microenvironment. CD44 promotes tumor progression through the activation of low molecular weight hyaluronan, which in turn activates signaling pathways promoting cell migration and invasion, or by acting as a co-receptor to oncogenes (c-Met and ErbB receptors). CD44 may also inhibit tumor progression by binding to high molecular weight hyaluronan and promote its interaction with hypophosphorylated Merlin, inhibit RAS activation, inhibit CD44–ERM interactions and suppress EGFR activation [63], [64]. Studies by Lakshman *et al*. [65] and Zhang *et* *al*. [22] have shown that by introducing CD44 in CD44 negative cells the AKT phosphorylation was reduced. Our findings confirms a previous study by Peng *et al.* in breast cancer cell-lines, transfected with myristoylated AKT (Myr-AKT) isoforms via retroviral delivery system, where AKT isoforms uniformly decreased the frequency of CD44 subpopulations [64]. We have further verified this interaction, with flow cytometry, western blot and mRNA expression analysis in the colon cancer cell-line DLD-1 by showing that knock-out of *AKT*1, *AKT*2 or both *AKT*1 and *AKT*2 increased the expression of CD44. AKT indeed had a large impact on the total CD44 expression, but we did not see any differences in the expression of several of the CD44 isoform variants. One explanation for this could be that all experiments were carried out under optimal cell growth conditions. Recent studies demonstrate that expression of CD44 variants increases with cellular stress like serum starvation [9]. The CD44 expression has previously been associated with AKT possibly via the LYN-pathway [23], [25]. We confirmed this association by showing that the mRNA expression of LYN was increased in the *AKT*1/2 KO. This was however not seen in the single *AKT*1 or *AKT*2 KO cell-lines.

We further evaluated the difference in *AKT*1 KO and *AKT*2 KO cell-lines with a gene expression analysis focusing on the EMT pathway. EMT is induced by the Notch and Wnt pathways and involves reduction in cell adhesion and increased cell migration. Previous studies have shown that AKT is involved in the EMT process and that the EMT transition is suppressed when AKT is activated or up-regulated [64]. It has also been shown that CD133/CD44 expressing colon cancer cells express EMT markers [19]. However, we show that there were no differences in the expression of the EMT markers between the DLD-1 isogenic *AKT* knock-out cell-lines. This indicates that AKT is not essential in the EMT pathway in the DLD-1 cell-line. On the other hand, genes in the cell adhesion pathway (ALCAM, CLDN1, ITGB8, NEO1 and PVRL3) were up-regulated in the *AKT*2 KO cell-line cell lines indicating that AKT2 may have a suppressing role in the cell adhesion. Two genes in the Notch pathway (*JAG1* and *HES)* had a lower expression in the *AKT*1 KO cell-line suggesting that AKT1 is involved in this pathway. There were two genes in the WNT/Ca2+-pathway (*PRKCA* and *WNT5A)*, which is independent of β-catenin, that were differently expressed. However, *PRKCA* was up-regulated and *WNT5A* was down-regulated in the *AKT*2 KO cell-line.

In summary, the three colon cancer cell lines had a varying expression of CD133, CD44 and CD24. However, in all three cell-lines the CD133/CD44 highly expressing cells were more resistant to radiation and had a higher expression of AKT. The knock-out of *AKT* also increased radiation sensitivity in DLD-1. We would have expected that the knock-out of *AKT* would also reduce the expression of CD133 and CD44, but instead showed that CD133 expression was only reduced in the *AKT*1 KO and *AKT*1/2 KO cell-lines, and that there was an increase in the CD44 expression in the *AKT* KO cell-lines. This would suggest that the use of an AKT inhibitor could indeed increase the radiation sensitivity but may instead induce CD44 expressing cancer cells. Since EMT is believed to be involved in cancer stem cells as well as AKT, the gene expression markers for this pathway were further analyzed. However there was no difference in the expression of EMT related genes between the *AKT*1 and *AKT*2 KO cell-lines. On the other hand the *AKT*2 KO cell line had a higher expression of genes involved in the cell adhesion.

## Conclusion

This study presents the association of CD133 and CD44 in terms of radiation resistance in colon cancer cell-lines. We demonstrate the importance of AKT and how its isoforms influence the expression of CD133 and CD44. The CD133 expression was reduced in the *AKT*1 KO but not *AKT*2 KO colon cancer cell line, whereas the expression of CD44 was increased by both *AKT*1 KO and *AKT*2 KO. Our findings suggest that combinations of inhibitors against AKT and CD44 could be used to avoid negative feed-back loops associated with AKT inhibitors which may cause the cancer cells to survive treatment.

## Supporting Information

Figure S1 **Clonogenic assay o DLD-1, HCT116 and HT-29.** Unsorted cells were exposed to 0, 2, 4 and 6 Gy of γ-irradiation.
(TIF)

Figure S2 **Confirmation of AKT1, AKT2, and AKT1/2 KO with RT-PCR.** RNA was extracted and RT-PCR was performed on DLD-1 parental, *AKT*1 KO, *AKT*2 KO and *AKT*1/2 KO cell-lines. The full-length wild-type (wt) AKT1 and AKT2 are marked with an arrow.
(TIF)

Figure S3 **DLD-1 parental cells transfected with siRNA against AKT1.** The expression of CD44, CD133 and CD24 were analyzed with flow cytometry 48 hours after transfection. The siAKT1 transfected cells show a reduction is CD133 expression from 16% to 10% (37.5% change).
(TIF)

Figure S4 **Reintroduction of AKT1 and AKT2 in DLD-1 AKT1/2 KO.** pcDNA3.0 plasmid with Myr-AKT1 or Myr-AKT2 were transfected in DLD-1 *AKT*1/2 KO cells. The expression of CD44, CD133 and CD24 were analyzed with flow cytometry. The pcDNA3-Myr-AKT1 and Myr-AKT2 transfected cells show a small population with lower CD44 expression, 3.5 and 4.5% respectively, compared to DLD-1 *AKT*1/2 KO cells. There was also an increase in CD24 from 0.2% in *AKT*1/2 KO to 2% and 5.5% in myr-AKT1 and myr-AKT2 cells.
(TIF)
