# Supplementary figures and images for: Evaluation of Cancer Stem Cell Markers CD133, CD44, CD24: Association with AKT Isoforms and Radiation Resistance in Colon Cancer Cells
Source: PLoS One. 2014 Apr 23;9(4):e94621. doi: 10.1371/journal.pone.0094621 (PMC3997403; doi:10.1371/journal.pone.0094621)

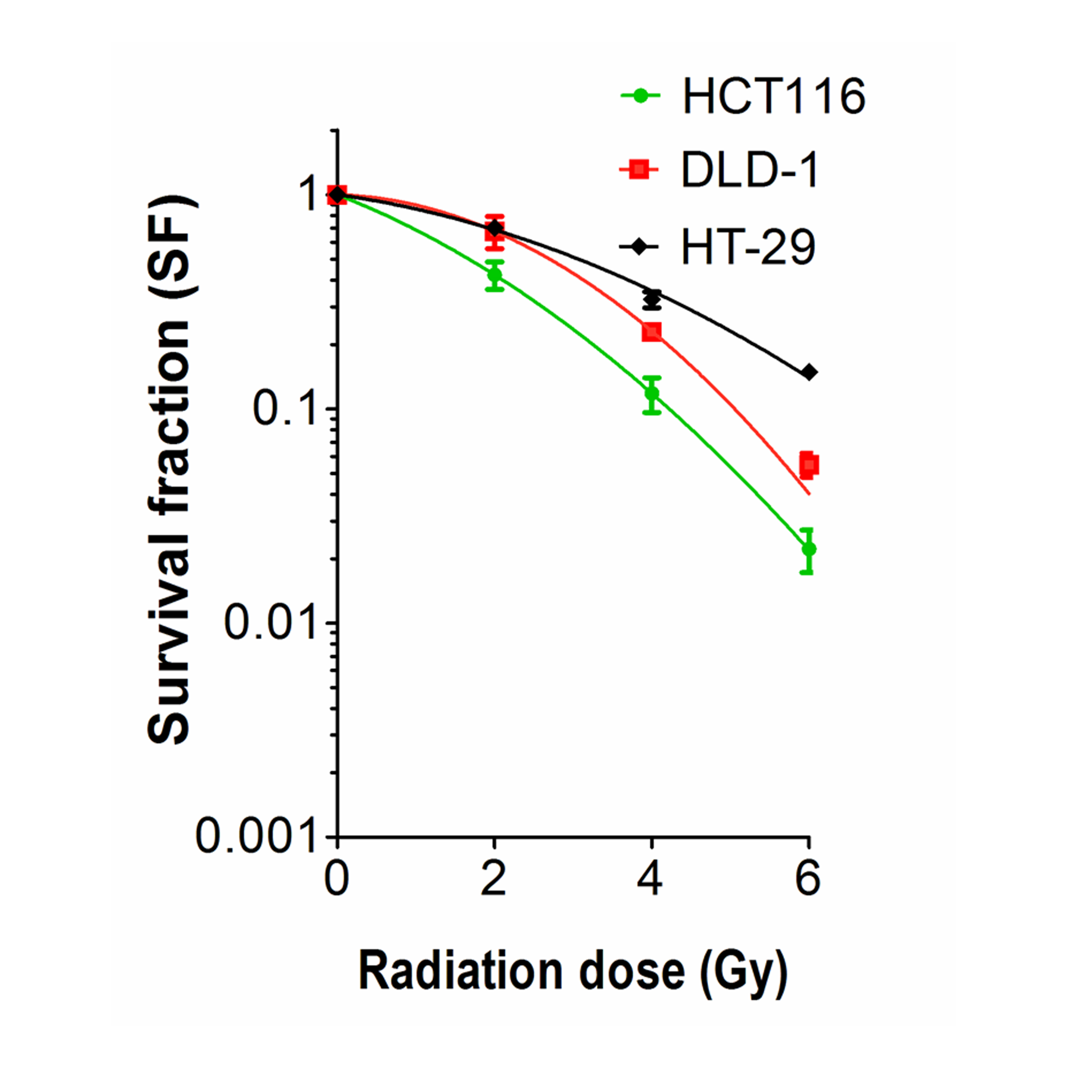

Supplement: Figure S1 — Clonogenic assay o DLD-1, HCT116 and HT-29. Unsorted cells were exposed to 0, 2, 4 and 6 Gy of γ-irradiation. (TIF) [file pone.0094621.s001.tif]

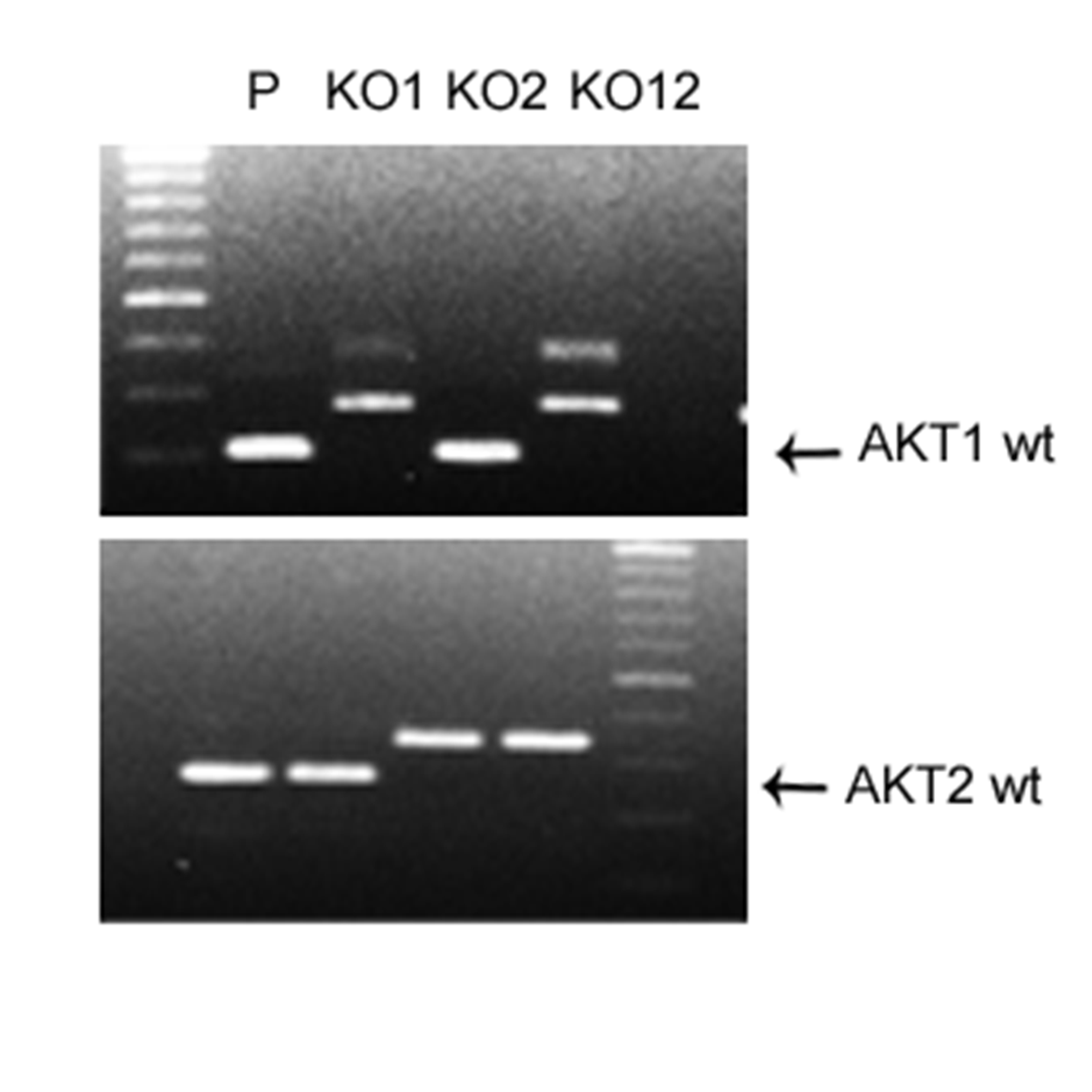

Supplement: Figure S2 — Confirmation of AKT1, AKT2, and AKT1/2 KO with RT-PCR. RNA was extracted and RT-PCR was performed on DLD-1 parental, AKT1 KO, AKT2 KO and AKT1/2 KO cell-lines. The full-length wild-type (wt) AKT1 and AKT2 are marked with an arrow. (TIF) [file pone.0094621.s002.tif]

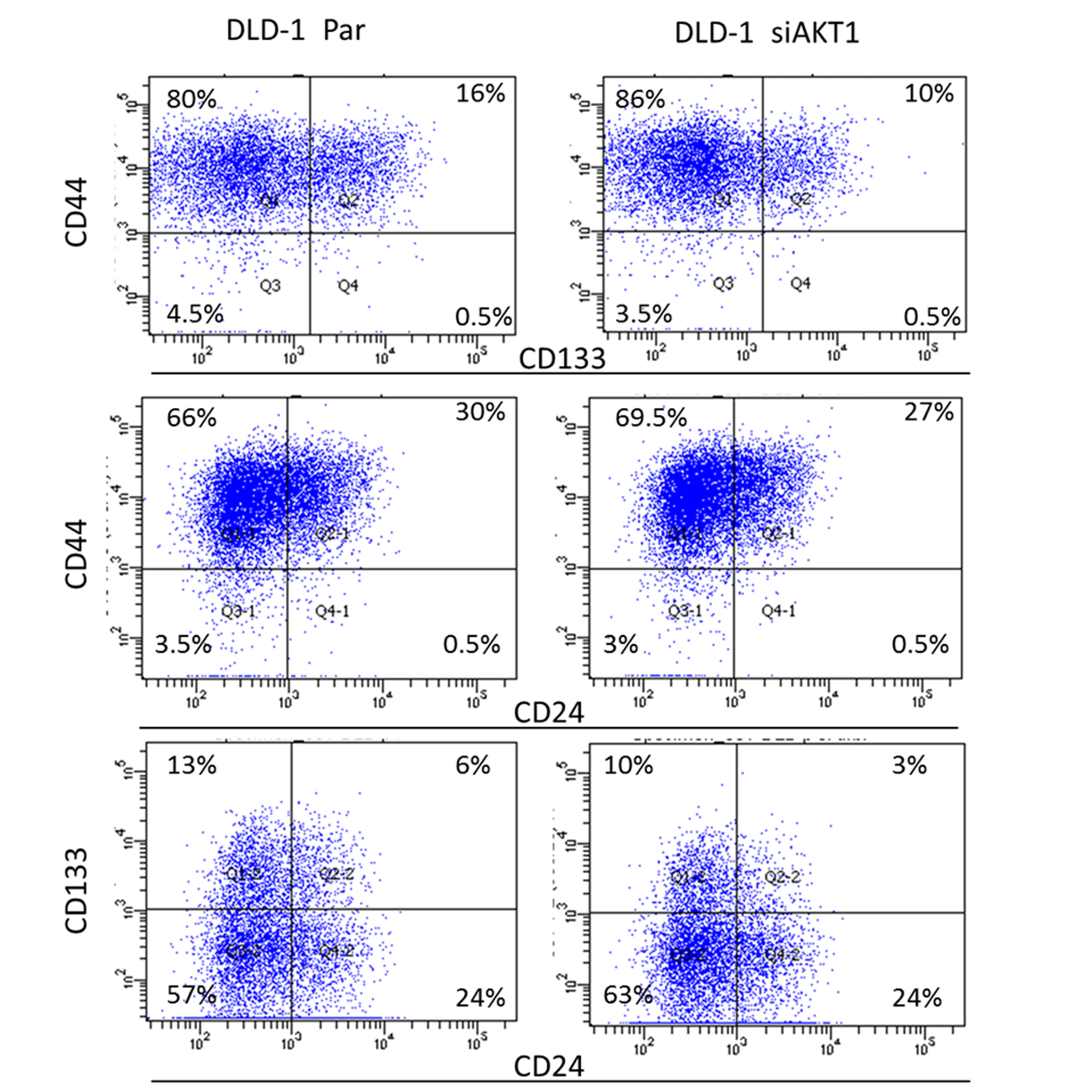

Supplement: Figure S3 — DLD-1 parental cells transfected with siRNA against AKT1. The expression of CD44, CD133 and CD24 were analyzed with flow cytometry 48 hours after transfection. The siAKT1 transfected cells show a reduction is CD133 expression from 16% to 10% (37.5% change). (TIF) [file pone.0094621.s003.tif]

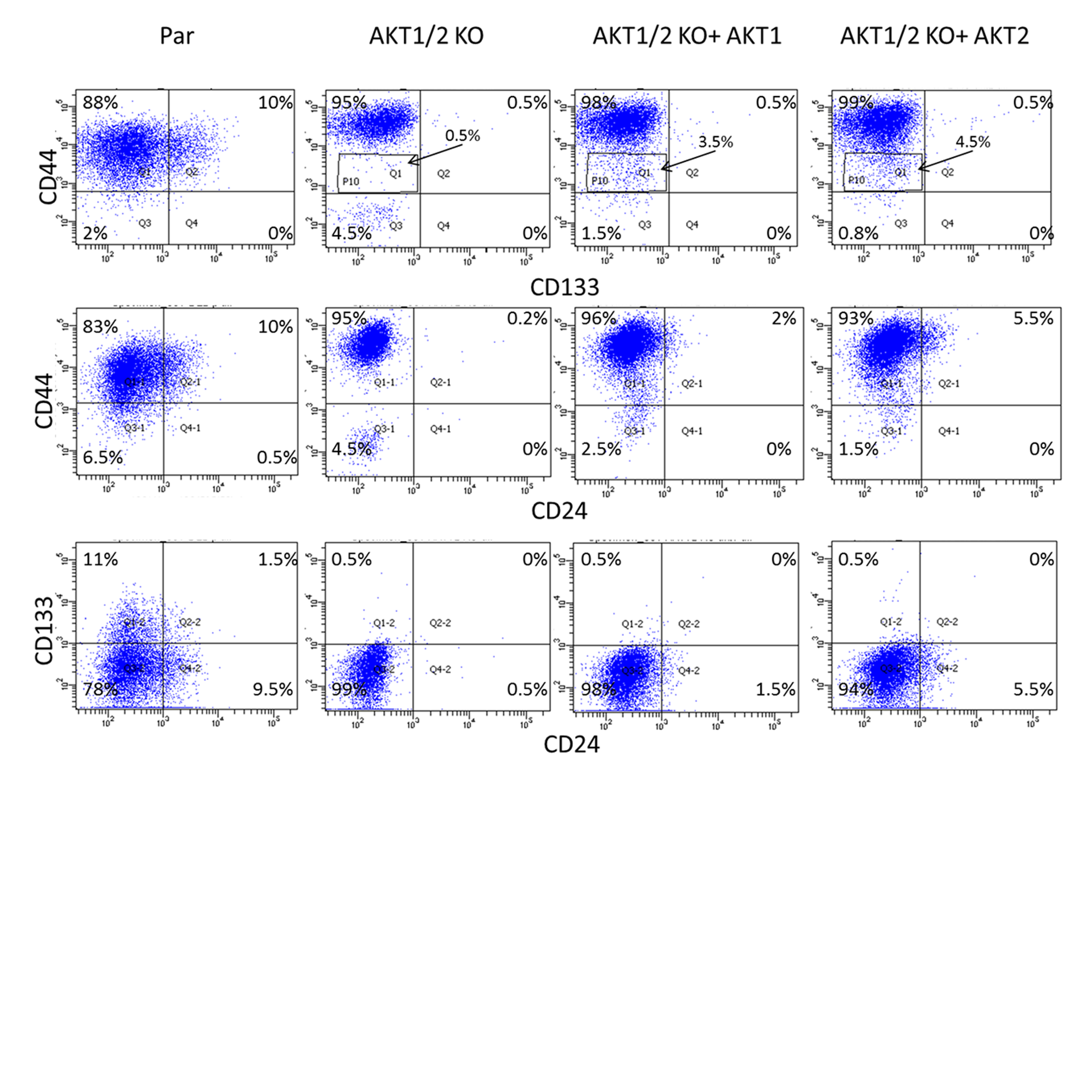

Supplement: Figure S4 — Reintroduction of AKT1 and AKT2 in DLD-1 AKT1/2 KO. pcDNA3.0 plasmid with Myr-AKT1 or Myr-AKT2 were transfected in DLD-1 AKT1/2 KO cells. The expression of CD44, CD133 and CD24 were analyzed with flow cytometry. The pcDNA3-Myr-AKT1 and Myr-AKT2 transfected cells show a small population with lower CD44 expression, 3.5 and 4.5% respectively, compared to DLD-1 AKT1/2 KO cells. There was also an increase in CD24 from 0.2% in AKT1/2 KO to 2% and 5.5% in myr-AKT1 and myr-AKT2 cells. (TIF) [file pone.0094621.s004.tif]
